# Elevated expression of Par3 promotes prostate cancer metastasis by forming a Par3/aPKC/KIBRA complex and inactivating the hippo pathway

**DOI:** 10.1186/s13046-017-0609-y

**Published:** 2017-10-10

**Authors:** Pei-Jie Zhou, Wei Xue, Jinliang Peng, Yanqing Wang, Lianzi Wei, Ziqiang Yang, Helen He Zhu, Yu-Xiang Fang, Wei-Qiang Gao

**Affiliations:** 1 State Key Laboratory of Oncogenes and Related Genes, Renji-MedX Clinical Stem Cell Research Center, Ren Ji Hospital, School of Medicine, Shanghai Jiao Tong University, 160 Pujian Road, Shanghai, 200127 China; 2 School of Biomedical Engineering & Med-X Research Institute, Shanghai Jiao Tong University, Shanghai, 200030 China; 3 Collaborative Innovation Center of Systems Biomedicine, Shanghai Jiao Tong University, Shanghai, 200240 China; 4 Department of Urology, Ren Ji Hospital, School of Medicine, Shanghai Jiao Tong University, Shanghai, 200127 China; 5 School of Pharmacy, Shanghai Jiao Tong University, Shanghai, 200240 China

**Keywords:** Par3, KIBRA, Hippo-YAP pathway, Prostate cancer, Metastasis

## Abstract

**Background:**

Prostate cancer (PCa) is one of the most frequent tumors and leading cause of cancer deaths among males worldwide. The majority of deaths are due to recurrence and subsequent development of the metastatic cancer. Although loss or dislocalization of polarity proteins has been implicated in embryogenesis deficiency and tumorigenesis, association of polarity protein expression levels with tumor metastasis remains unclear.

**Methods:**

Bioinformatics, qRT-PCR, western blot and immunohistochemical (IHC) analyses were used to examine expression of Par3, a key component of polarity-associated partitioning defective (PAR) complex, in primary and metastatic clinical PCa samples. Loss-of-function and gain-of-function studies in vitro and in vivo were performed to determine the functions of Par3 during metastasis of PCa. Co-immunoprecipitation (co-IP), western blot, immunofluorescence (IF), chromatin immunoprecipitation (ChIP) and qRT-PCR analyses were conducted to investigate the underlying mechanism for the function of Par3 on PCa metastasis.

**Results:**

In this study, we found that elevated expression of Par3 is positively associated with PCa metastasis. Knockdown of Par3 inhibits PCa cell migration and invasion in vitro and tumor metastasis in vivo, whereas overexpression of Par3 yields the opposite results. Mechanistically, Par3 suppresses phosphorylation of LATS to inactivate the Hippo pathway and enhances nuclear translocation of YAP by sequestrating KIBRA from the KIBRA/Merlin/FRMD6 complex and forming a Par3/aPKC/KIBRA complex. Stable knockdown of Par3 leads to restoration of the KIBRA/Merlin/FRMD6 complex and activation of the Hippo pathway, and then results in an inhibition on YAP nuclear translocation. In addition, in conjunction with the TEA domain (TEAD) transcription factor family, intranuclear YAP promotes the transcription of several pro-metastatic genes such as the matrix metalloproteinase (MMP) family, Zeb1, Snail1 and Twist1. Moreover, knockdown of Par3 downregulates expression of these pro-metastatic genes.

**Conclusions:**

Our findings indicate that elevated expression of Par3 promotes PCa metastasis via KIBRA sequestration-mediated inactivation of the Hippo pathway to upregulate expression of pro-metastatic genes. Downregulation of Par3 expression may serve as a potential treatment approach for PCa metastasis by activating the Hippo pathway.

**Electronic supplementary material:**

The online version of this article (10.1186/s13046-017-0609-y) contains supplementary material, which is available to authorized users.

## Background

Prostate cancer (PCa) is one of the most frequent tumors and leading cause of cancer deaths among males in the world [1]. Although surgical treatment, androgen blockade or radiotherapy is available at the early stage of the disease, most patients sooner or later suffer from recurred tumors and metastasis which will destroy the bone marrow and results in death. Therefore, investigation of the molecular mechanisms involved in prostatic tumor progression, especially metastasis, is urgently needed to identify potential effective therapeutic targets to improve patient survival [2, 3].

Cell polarity is essential for epithelial cells to maintain their columnar shape and to exert their physiological functions. To date, three major evolutionarily conserved polarity complexes have been identified: the partitioning defective (PAR) complex including Par3, Par6 and atypical protein kinase C (aPKC), the crumbs complex and the scribble complex [4]. As most human solid tissue tumors arise from epithelial cells, aberrant cell polarity is assumed to be a common hallmark of epithelial cancers [4]. Recent studies by several groups suggest that dislocalization of polarity proteins, Par3 and scribble, for instance, are intricately related to early stages of tumorigenesis via different signal transduction pathways [5–7]. In addition, it has been revealed that dys-regulation of Par3 is also associated with tumor metastasis and poor prognosis [5, 8, 9]. However, the exact role of Par3 in PCa metastasis is still poorly understood and the underlying molecular mechanisms are unclear.

In the past a few years, special attention has been paid to a novel, highly conserved Hippo-YAP pathway for its essential role in tissue homeostasis and in tumor progression [10, 11]. The Hippo-YAP pathway consists of serine/threonine kinase Mst1/2, large tumor suppressor kinase 1/2 (LATS1/2) and the effector Yes-associated protein (YAP). Both phospho-LATS1/2 and phospho-Mst1/2 are active forms, which respond to the upstream signals and then induce the cytoplasmic location and degradation of phosphorylated YAP [12, 13]. Conversely, non-phosphorylated YAP is translocated into the nucleus and induces gene transcription by conjunction with the TEA domain family (TEAD) transcription factors [14]. Increasingly available evidence indicate that besides regulation of tumor growth [15, 16], the Hippo-YAP pathway is clinically involved in tumor metastasis. However, the underlying mechanism for the involvement of the Hippo-YAP pathway in metastasis is still needed to be elucidated [17–19]. On the other hand, investigation on upstream regulators of the Hippo-YAP pathway is still limited. Although several proteins such as G protein-coupled receptor (GPCR) [20], Merlin/NF2 [21], or Willin/FRMD6 [22], a homologue of Expanded in *Drosophila,* can act as either an activator or inhibitor of the Hippo-YAP pathway, whether there are other possible upstream regulators for this pathway in the mammalian cells, especially in human cancer cells are not determined.

In this study, we aimed to determine possible roles of Par3 in prostate cancer metastasis. We found that enhanced expression of Par3 in the tissues from PCa patients as well as PCa cell lines is positively associated with tumor metastasis. We also obtained evidence suggesting that Par3 acts as a potential upstream regulator of the Hippo-YAP pathway via sequestration of KIBRA, a reported activator of Hippo pathway [23], from canonical KIBRA/Merlin/FRMD6 complex [24] and formation of a Par3/aPKC/KIBRA complex to suppress phosphorylation of the Hippo pathway and YAP. Finally, by increasing nuclear translocation of non-phosphorylated YAP and activation of TEAD transcription factors, the transcription of pro-metastasis genes is enhanced, which promotes the PCa metastasis. Thus, repression of Par3 may offer a potential treatment approach to inhibit PCa metastasis by activating the Hippo pathway.

## Methods

### Cell lines and cell culture

Prostate cancer cell lines PC3, DU145 and normal prostate epithelial cell line PNT1B were purchased from the American Type Culture Collection (ATCC, Rockville, MD, USA). Cells were cultured in Dulbecco’s modified Eagle’s medium (DMEM, Gibco, Thermo Fisher Scientific, Waltham, MA, USA) with 10% fetal bovine serum (FBS, Gibco) and maintained at 5% CO_2_ at 37 °C.

### Cell transfection and lentiviral vector infection

The Par3 knock down and control plasmids were obtained from Origene (Rockville, MD, USA). Plasmids were transfected with Lipofectamine3000 (Thermo Fisher Scientific). Puromycin (0.5 μg/ml) was used for selecting stable Par3 knockdown and relevant control subclones, which were named as PC3-shPar3, PC3-con, DU145-shPar3 and DU145-con respectively. The lentiviral vector expressing one of the Par3 isoforms (NM_019619.3, 150 kDa) or a non-phosphorylatable YAP mutant, YAP(S127A); and control lentiviral vector were constructed and packaged by Genomeditech Comp (Shanghai, China). 2 × 10^6^ cells were seeded in 6-well plates and infected using relevant lentiviral vector (MOI = 10 for each) concomitant with 5 μg/ml polybrene. Western blot was employed to detect Par3 expression levels. IF was employed to detect YAP subcellular location. MOI: multiplicity of infection.

### In vitro migration and invasion assay

In vitro migration and invasion assays were performed using 24-well Cell Migration and Invasion Assay kit (Cell BioLabs, San Diego, CA, USA), according to the manufacturer’s instructions. Briefly, after serum starvation for 24 h, 1 × 10^5^ cells were suspended in 100 μl DMEM basic medium and seeded in the upper chamber, and 700 μl DMEM medium with 10% FBS was added to the lower chamber. After incubation for 6 h (for migration assay) or 8 h (for invasion assay), cells on the lower surface of the membrane were fixed with 4% paraformaldehyde, stained with 0.2% crystal violet, photographed and counted under a microscope in five random fields.

### Orthotopic transplantation of PCa cell lines

Six-week-old male BALB/C athymic nude mice (SLAC, Shanghai, China) were housed and manipulated according to the protocols approved by the Renji Hospital Medical Experimental Animal Care Commission. For orthotopic inoculation, 1 × 10^6^ cells were first injected under the subcutaneous and tumor mass was harvested after 4 weeks. Xenografts were digested with collagenase IV for 30mins, 0.05% Trypsin for 10mins and then normalized by DMEM medium with 10% FBS to collect cells for orthotopic transplantation. Cell suspension (1 × 10^5^ cells in 20 μl) was mixed with 20 μl matrigel and injected into the left ventral anterior of mouse prostate. PET-CT (Siemens Inveon) detection was performed using ^18^F–FDG (750 μCi/100 g) by intravenous injection 7 weeks after orthotopic inoculation. Orthotopic tumor growth and potential tumor metastasis were evaluated in living animals by the absorption of ^18^F–FDG. Mice were sacrificed 1 week after PET-CT detection. Orthotopic tumor mass and metastatic nodes were collected for H&E and immunofluorescence staining.

### Clinical samples

Investigation has been conducted in accordance with the ethical standards and according to the Declaration of Helsinki and national and international guidelines. Human tissues used in this study were reviewed and approved by the Committee for Ethical Review of Research Involving Human Subjects at Renji Hospital. PCa (*n* = 14 for qRT-PCR and western blot, *n* = 2 for immunohistochemical) and normal tissues (*n* = 7 for qRT-PCR and western blot, n = 1 for immunohistochemical) were obtained from the Renji Biobank, Shanghai Jiao Tong University School of medicine [25]. Written informed consent was obtained from all patients.

### H&E staining, Immunohistochemical (IHC) and Immunofluorescence (IF) staining and microscopy

Cells were seeded on cover slide placed in 24-well plate and cultured in DMEM medium supplemented with 10% FBS and maintained at 5% CO_2_ at 37 °C for 48 h. Adherent cells on cover slide were then fixed in 4% paraformaldehyde for 30mins at room temperature. Cells were washed with PBS and blocked with 10% normal goat serum (Vector, Burlingame, CA, USA) for 1 h at room temperature for IF staining. Tissues were fixed with 4% paraformaldehyde for 24 h and embedded in paraffin. H&E staining was performed by Runnerbio biotech. Comp (Shanghai, China). Paraffin sections were dewaxed in xylene for 5 min, sequentially hydrated in ethanol with concentrations of 100%, 95%, 85% and 70% for 3 min each, respectively, and rinsed three times in water. Sections were treated with disodium-hydrogen phosphate-2-hydrate for 15 min to inactivate endogenous peroxidase and then blocked with 10% normal goat serum for 1 h at room temperature for IF and IHC staining. After serum blocking, both cells and sections were incubated with relevant primary antibodies (1:200, diluted in PBS with 1% normal goat serum) overnight at 4 °C. For IF staining, cells and sections were washed with PBS for three times (10mins each) and then incubated with Alexa Fluor-546 or Alexa Fluor-488 conjugated secondary antibody (Thermo Fisher Scientific) for 1 h at room temperature. Cells and sections were washed with PBS for three times again before mounted with DAPI containing mounting medium (Vector). For IHC staining, sections were washed with PBS for three times (10 min each) and then incubated with horseradish peroxidase-conjugated secondary antibody (Vector) for 1 h at room temperature. Sections were washed with PBS for three times again before DAB staining (Sangon Biotech, Shanghai, China) and hematoxylin counterstaining (Beyotime, Shanghai, China). Primary antibody for YAP (#4912) was purchased from Cell Signaling Technology (Beverly, MA, USA) and for Par3 (07–330) and human nuclei (MAB1281) from Millipore (Billerica, MA, USA). IF and IHC staining was visualized under a microscope (Leica DFC420C) and images were merged using the ImageJ software.

### RNA isolation and qRT-PCR analysis

Total RNA was extracted using Trizol (Thermo Fisher Scientific) according to the method previously described [26]. For mRNA relative expression analysis, total RNA was reversed transcribed into cDNA with Prime-Script RT kit (Takara, Shiga, Japan) and amplified with SYBR-Green Real-time PCR Master Mix (Applied Biosystems, Thermo Fisher Scientific). The mRNA expression level of GAPDH was used as an internal normalization control. Comparative quantification was performed by using the 2^-ΔΔCt^ method. All primers are available in the Additional file 1: Table S1.

### Western blot and co-immunoprecipitation (co-IP)

For western blot, 1 × 10^7^ cultured cells were lysed with nuclear and cytoplasmic protein extract kit (Cat. No. PP1082, Bioteke, Shanghai, China) or membranous and cytoplasmic protein extract kit (Thermo Fisher Scientific) supplemented with a Complete Protease Inhibitor Cocktail and a final concentration of 2 mM PMSF (Thermo Fisher Scientific) to isolate and purify nuclear vs. cytoplasmic or membrane vs. cytoplasmic protein respectively. Clinical normal or tumor tissues were lysed with RIPA (Millipore). The concentration of total proteins was measured by BCA (bicinchoninic acid) kit (Thermo Fisher Scientific) respectively. Forty micrograms of total proteins from relevant samples above were separated by 10% SDS-PAGE and then transferred to polyvinylidene fluoride membrane (Millipore) under the same experimental condition. The membrane was blocked with TBST containing 5% BSA for 1 h, incubated with specific primary antibodies overnight at 4 °C and then probed with HRP-conjugated secondary antibody at room temperature for 1 h. Proteins were detected with HRP Substrate (Millipore) and photographed using ECL (electro-chemiluminescence) detection instrument (Thermo Fisher Scientific).

For immunoprecipitation, 1 × 10^7^ cultured cells were lysed with RIPA (Millipore) lysate and 100 μg total protein were incubated on ice with 5 μg specific antibodies and 40 μl protein G-sepharose beads (Thermo Fisher Scientific) overnight. Beads-antibody complex was washed three times with Chilled PBS and boiled for 10 min to obtain protein supernatant captured by the protein G-sepharose beads. Antibodies for western blot and co-IP analysis are listed in the Additional file 1: Table S2.

### Chromatin immunoprecipitation (ChIP) assays

Cell extraction was prepared using Chromatin IP kit (Cell Signaling Technology) according to the manufacturer’s protocol. Briefly, 1 × 10^7^ cells were cross-linked with 37% formaldehyde, collected and digested to produce chromatin fragments for incubation with IgG (Cell Signaling Technology) or specific antibodies for Tead1 (ab133533, Abcam, Cambridge, MA, USA), Tead2 (H00008463-M01A, Abnova, Taipei, Taiwan), Tead4 (ab58310, Abcam) and YAP (#4912, Cell Signaling Technology) respectively. ChIP DNA was amplified and analyzed by qPCR and sequencing. Relative enrichment of specific factors was assessed by the formula provided in the protocol. ChIP primers are listed in the Additional file 1: Table S3.

### Statistical analysis

Independent Student’s *t*-test and analysis of variance (ANOVA) were used to compare the differences between two groups. Difference of Par3 expression between normal and PCa tissues was analyzed using Prism GraphPad5 (GraphPad Software, La Jolla, CA, USA). All data were represented as mean ± SD from triplicate experiments. Results were considered statistically significant when *p* < 0.05.

## Results

### Par3 expression is markedly elevated in clinical metastatic prostate cancer tissues

**Fig. 1:**
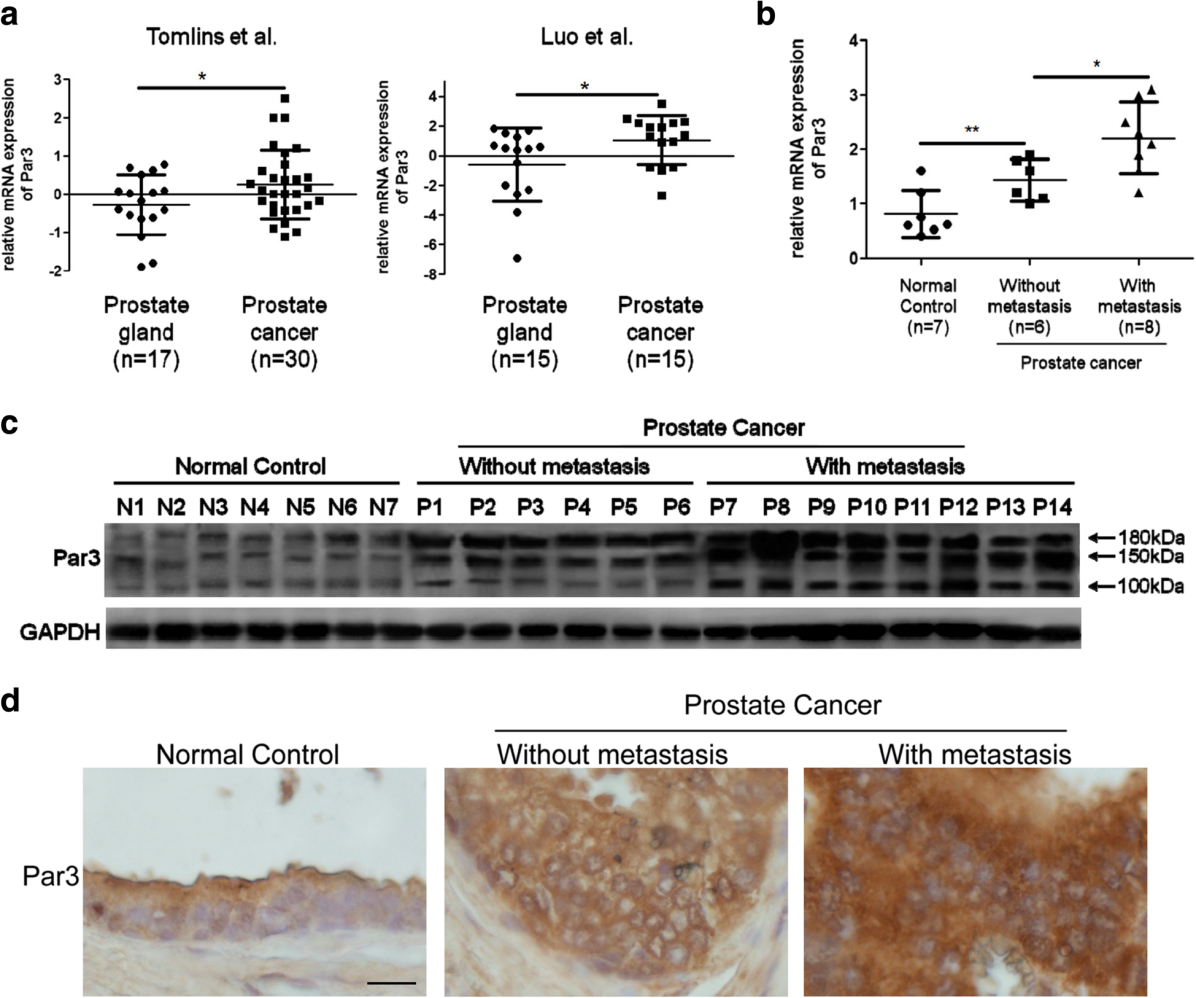
Par3 expression is upregulated in metastatic PCa tissues. **a** Data from Oncomine database demonstrates a significant upregulation of Par3 expression in PCa tissues compared to normal controls. **b**, **c** Expression of Par3 at mRNA (**b**) and protein levels (**c**) in clinical samples from patients with or without metastasis and in that from normal persons. N: normal control, P: patient. **d** Expression of Par3 in clinical samples by IHC staining. Scale Bar: 20 μm. All data are represented as mean ± SD from triplicate experiments. *: *p* < 0.05, **: *p* < 0.01

In order to investigate the relationship between Par3 expression and PCa, we analyzed two independent datasets from Oncomine database (Tomlins et al. [27] and Luo et al. [28]). Both of the datasets exhibited a significant upregulation of Par3 expression in PCa tissues compared to normal controls, indicating that enhanced expression of Par3 is positively associated with PCa (Fig. 1a). Consistently, analysis of the TCGA PRAD database also revealed that Par3 expression level was elevated in PCa tissues (Additional file 2: Figure S1). To determine whether expression of Par3 is further upregulated in metastatic prostate tumors, we compared Par3 expression in primary tumor tissues derived from patients with (*n* = 8) or without metastasis (*n* = 6) to that in prostatic tissues (*n* = 7) derived from normal population at both mRNA and protein levels (Fig. 1b, c, Additional file 3: Table S1). We found that expression of Par3 was enhanced in all tumor samples compared to normal tissues. In addition, expression of Par3 was further enhanced in tumor tissues from patients with metastasis, indicating a positive correlation of Par3 expression with metastasis (Fig. 1b, c). By IHC staining in clinical samples, we confirmed that expression of Par3 was enhanced in tumor tissues especially in those with metastasis (Fig. 1d, Additional file 3: Table S2). These observations together indicate that enhanced expression of Par3 is positively correlated with human PCa metastasis.

### Knockdown of Par3 inhibits PCa cell migration in vitro and metastasis in vivo

In order to provide direct experimental evidence that Par3 regulates PCa metastasis, we performed a Par3 knockdown experiment in PCa cell lines. For this purpose, we first measured endogenous expression of Par3 in normal prostatic cell lines PNT1B and PCa cell lines PC3 and DU145. We found that compared to PNT1B cells, Par3 expression was significantly elevated in PC3 and DU145 cells, both of which possess migratory capability (Additional file 2: Figure S2a). Thus we established Par3 stable knockdown subclones or controls with these two cell lines respectively to evaluate the effect of Par3 on migration in vitro and metastasis in vivo (Additional file 2: Figure S2b, Figure S2c). In addition, we also established Par3-overexpressing PC3 cells (PC3-Par3OE) for further confirmation of relevant observations (Additional file 2: Figure S2d).

**Fig. 2:**
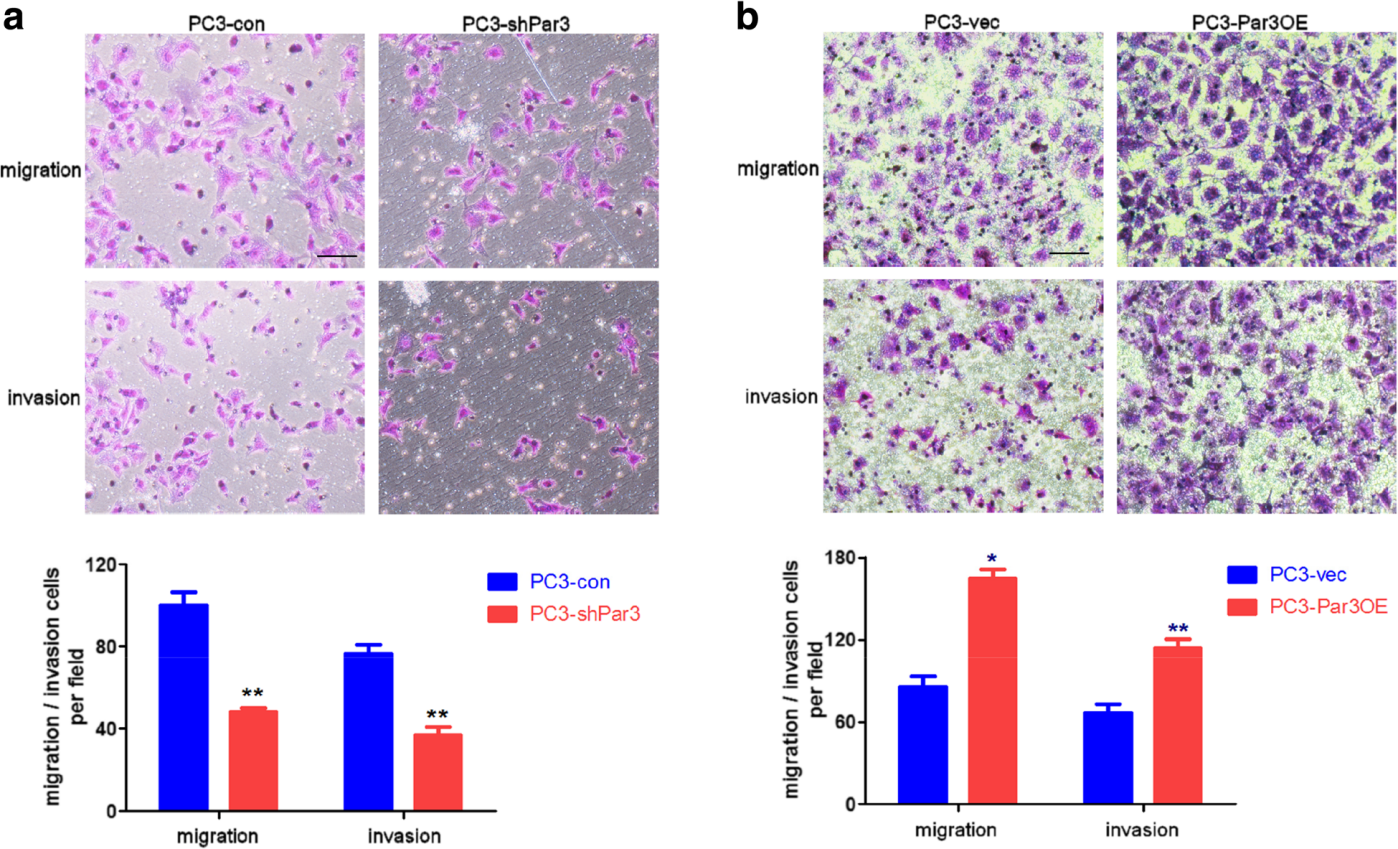
Par3 knockdown inhibits while Par3 overexpression facilitates PC3 cell migration and invasion in vitro. **a** Stable knockdown of Par3 inhibits both migration and invasion in PC3 cells by transwell assays. Migratory and invasive cells are counted and averaged from five randomly selected fields respectively. Scale Bar: 20 μm **b** Overexpression of Par3 activates both migration and invasion in PC3 cells by transwell assays. Migratory and invasive cells are counted and averaged from five randomly selected fields respectively. Scale Bar: 20 μm. All data are represented as mean ± SD from triplicate experiments. *: *p* < 0.05, **: *p* < 0.01

To determine whether downregulation of Par3 affects PCa cell migration and invasion in vitro, we performed transwell assays. As shown in Fig. 2a **,** the migratory capability was decreased to about 50% in PC3 (50.6 ± 3.36 cells/field in PC3-shPar3 vs 103.4 ± 8.68 cells/field in PC3-con) and to about 46% in DU145 (42.6 ± 6.95 cells/field in DU145-shPar3 vs 92.2 ± 13.71 cells/field in DU145-con) after Par3 knockdown respectively (Fig. 2a, Additional file 2: Figure S3). The invasive capability was also decreased to about 48% in PC3 (37.8 ± 5.57 cells/field in PC3-shPar3 vs 78.1 ± 7.42 cells/field in PC3-con) and to about 38% in DU145 (24.4 ± 5.95 in DU145-shPar3 vs 64.2 ± 5.73 in DU145-con) after Par3 knockdown respectively (Fig. 2a, Additional file 2: Figure S3). Conversely, after overexpession of Par3 in PC3 cells by lentiviral infection, both migratory and invasive capabilities were improved more than 1.5 folds (Fig. 2b). These results together indicate that stable Par3 knockdown inhibits the migration and invasion of PCa cells in vitro.

**Fig. 3:**
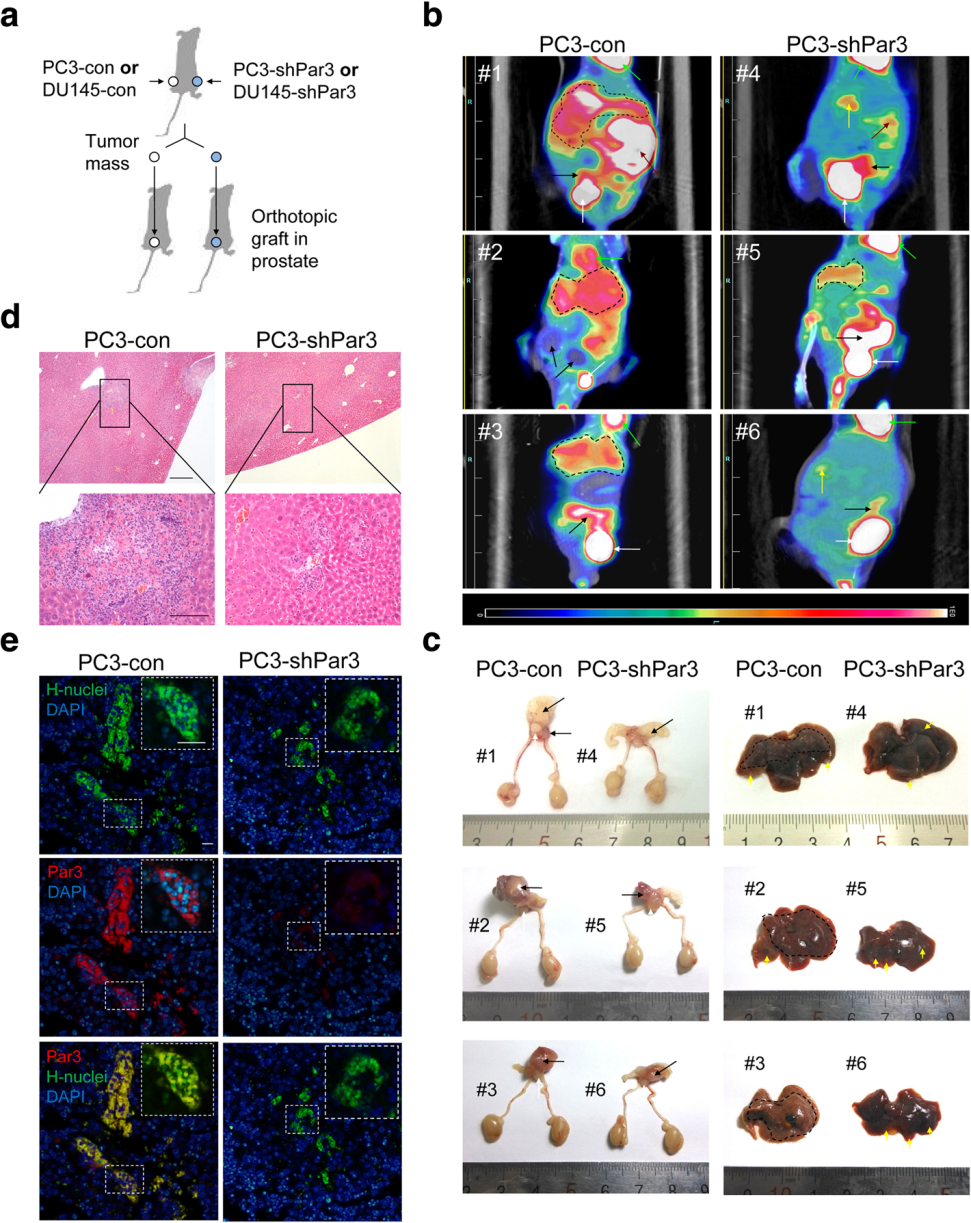
Knockdown of Par3 inhibits PCa metastasis in vivo. **a** Strategy for evaluating the effect of Par3 knockdown on metastasis in vivo. **b** Representative PET-CT images from orthotopic implantation mouse models by inoculation of PC3-con cells (#1-#3, *n* = 3) or PC3-shPar3 cells (#4-#6, *n* = 3). White arrow: bladder, black arrow: orthotopic tumor, dark red arrow: invasion in peritoneal lymph nodes, yellow arrow and field in black broken line: metastatic nodes in liver, bright green: heart. **c** Orthotopic grafts and liver metastasis are dramatically regressed in PC3-shPar3 inoculated mouse (#4-#6) than control (#1-#3). White arrow: bladder, black arrow: orthotopic tumor, yellow arrow and field in black broken line: metastatic nodes in liver. **d** H&E staining of liver tissues from orthotopic PC3-shPar3 or PC3-con cells inoculated mice. Field in black broken line and frame: metastatic nodes. Scale Bar: 100 μm for upper panels; 50 μm for lower panels. **e** IF staining of liver tissues for Par3 expression from orthotopic PC3-shPar3 or PC3-con cells inoculated mice. H-nuclei: human nuclei. Scale Bar: 20 μm

To further confirm whether Par3 knockdown leads to an inhibition on metastasis of PCa cells in vivo, we conducted orthotopic implantation experiments using PC3-shPar3 vs PC3-con cells and DU145-shPar3 vs DU145-con cells. Relevant cells were first inoculated subcutaneously in 6-week old male BALB/C athymic nude mice and then dissociated from tumor mass 4 weeks later prior to orthotopic implantation via injection of 1 × 10^5^ cells into the left anterior of prostate (Fig. 3a). Orthotopic tumor growth and micro- or macro-metastases were evaluated in living animals by PET-CT 7 weeks post-implantation. We found a significant inhibition on distant liver metastasis after orthotopic implantation of PC3-shPar3 (#4 to #6) compared to PC3-con cells (#1 to #3, Fig. 3b). Serious invasion to peritoneal lymph nodes was also observed in one of the mice after inoculation of PC3-con cells but not PC3-shPar3 cells (#1 vs. #4 in Fig. 3b and Additional file 2: Figure S4a). The paravertebral lymph nodes invasion and kidney metastasis were found to be inhibited when using DU145-shPar3 cells for orthotopic implantation (Additional file 2: Figure S4b). Mice were sacrificed 1 week after PET-CT analysis and the orthotopic tumor growth, lymph nodes invasion and metastasis in the liver or kidney were examined and then confirmed by H&E staining (Fig. 3c, d, Additional file 2: Figure S4c). Orthotopic grafts were dramatically larger along with tissue adhesions and necrosis after inoculation of PC3-con or DU145-con cells (#1 to #3 in Fig. 3c left panel and #1, #2 in Additional file 2: Figure S4c) but restricted in the anterior prostate after inoculation of PC3-shPar3 or DU145-shPar3 cells (#4 to #6 in Fig. 3c left panel and #3, #4 in Additional file 2: Figure S4c), indicating that stable knockdown of Par3 inhibits orthotopic tumor growth. A large number of micro-metastatic nodes with several large ones were observed not only on the surface but also inside of the liver in PC3-con cells inoculated mice (Fig. 3c right panel #1 to #3, Fig. 3d), but only a limited number of nodes were found in PC3-shPar3 groups (Fig. 3c right panel #4 to #6, Fig. 3d). We further compared Par3 expression in metastatic nodes in livers of PC3-shPar3 to that of PC3-con inoculated mice, by co-staining Par3 with anti-human nuclei antibody to distinguish the metastatic human PCa cells from mouse liver cells. While maintained in those from control, Par3 expression was significantly suppressed in the liver metastatic nodes from PC3-shPar3 inoculated mice (Fig. 3e). Collectively, these results indicate that knockdown of Par3 significantly inhibits PCa cell migration in vitro and metastasis in vivo.

### Elevated expression of Par3 promotes PCa metastasis by inactivation of the hippo pathway

**Fig. 4:**
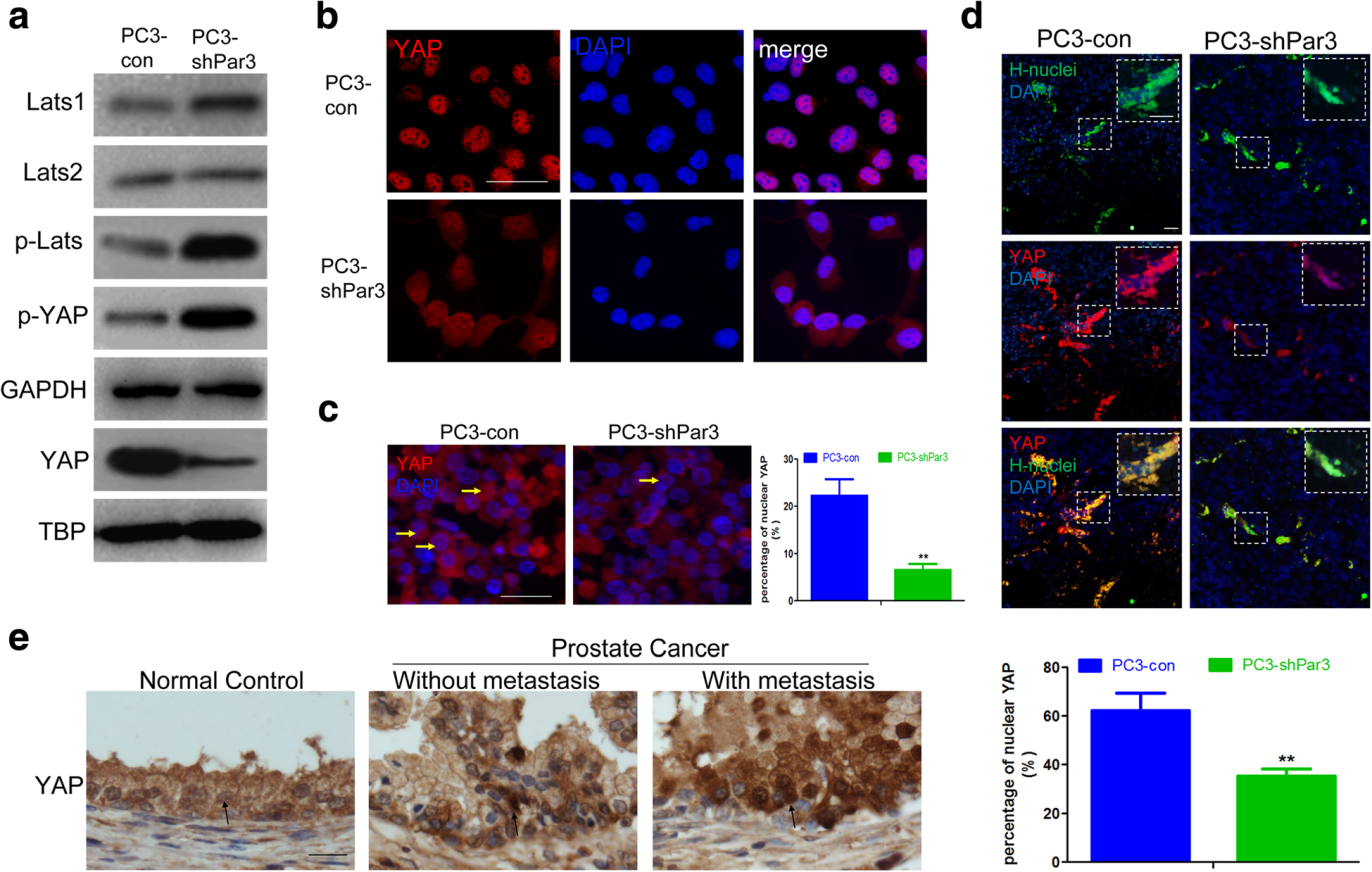
Knockdown of Par3 decreases the nuclear translocation of the oncogene YAP. **a** Knockdown of Par3 in PC3 cells enhances the phosphorylation of LATS and YAP so to suppress nuclear translocation of YAP. GAPDH: internal control for cytoplasmic proteins; TBP: internal control for nuclear proteins. **b** IF staining of nuclear translocation of YAP in PC3-con and PC3-shPar3 cells respectively in vitro. **c** Nuclear translocation of YAP is impaired in PC3-shPar3 vs. PC3-con cells inoculated orthotopic grafts respectively in vivo. Typical cells are indicated by yellow arrows. **d** YAP expression in liver tissues from orthotopic PC3-shPar3 or PC3-con cells inoculated mice. H-nuclei: human nuclei. **e** Expression of YAP in clinical samples by IHC staining. Typical cells are indicated by arrows. Scale Bar: 20 μm

Given that Hippo-YAP pathway has been reported to be involved in tumor metastasis [24], we herein suspect that Par3 might exert its effects on tumor metastasis via the Hippo-YAP pathway as a novel candidate upstream regulator. To provide evidences for this possibility, we investigated expression of LATS1/2, the major component of the Hippo-YAP pathway, in PC3-shPar3 cells and made a comparison to that in PC3-con cells. We found that after stable knockdown of Par3, expression levels of LATS1/2 were similar to that of the control group, but the level of phosphorylated LATS was significantly increased in PC3-shPar3 cells, indicating an activation of the Hippo pathway (Fig. 4a). As expected, the phosphorylation of YAP was consequentially enhanced (Fig. 4a) and its arrest in cytoplasm was increased in PC3-shPar3 cells (Fig. 4b). As a result, decreased nuclear localization of YAP was observed in PC3-shPar3 cells in vitro (Fig. 4b) as well as in PC3-shPar3 cells derived orthotopic grafts in vivo compared to relevant controls respectively (Fig. 4c). Expressional level of intranuclear YAP was also significantly decreased in liver metastatic nodes derived from PC3-shPar3 inoculated mice compared to the control mice (Fig. 4d). By IHC staining, we also observed that nuclear translocation of YAP was enhanced in tumor tissues, especially in those with metastasis (Fig. 4e, Additional file 3: Table S2). All these findings indicate that knockdown of Par3 inhibits PCa metastasis via activation of the Hippo pathway and antagonizing YAP through phosphorylation and arrest of YAP in the cytoplasm. To provide additional support for this notion, we also compared expression of LATS1/2 and the activation of LATS and YAP in PC3-Par3OE cells with the control cells. We found that forced overexpression of Par3 still had no effect on LATS1/2 expression but repressed the phosphorylation of both LATS and YAP, and eventually enhanced the nuclear translocation of YAP (Additional file 2: Figure S5a). Similar expression patterns of LATS1/2, phosphorylated LATS and intranuclear YAP were observed in DU145-shPar3 vs DU145-con cells in vitro (Additional file 2: Figure S5b,c), suggesting again that knockdown of Par3 can inhibit PCa cell migration in vitro and metastasis in vivo, associated with activation of the Hippo pathway.

In order to further determine whether knockdown of Par3 inhibits PCa metastasis by activating the Hippo pathway and attenuating the nuclear translocation of non-phosphorylated YAP as a response, we carried out a rescue experiment by exogenous overexpression of YAP(S127A), a non-phosphorylatable YAP mutant [29], in PC3-shPar3 cells to evaluate its effect on restoration of metastasis against Par3 knockdown (Additional file 2: Figure S6a). As expected, after overexpression of YAP(S127A), the effect of Par3 knockdown on inhibition of primary tumor growth and liver metastasis was significantly reversed in vivo (Additional file 2: Figure S6b, c, d). Moreover, increased nuclear translocation of YAP was also observed in metastatic nodes in liver, indicating no response to the activation of Hippo pathway by Par3 knockdown (Additional file 2: Figure S6e). Taken together, our results revealed that elevated expression of Par3 promotes PCa metastasis by inactivation of Hippo pathway and enhanced nuclear translocation of oncogenic YAP.

### Elevated expression of Par3 inactivates the hippo pathway via formation of a Par3/aPKC/KIBRA complex

In order to demonstrate how knockdown of Par3 activates the Hippo pathway, we paid attention to KIBRA, which was recently identified as an activator of the Hippo pathway by forming a KIBRA/Merlin/FRMD6 complex to interact with and sequentially to phosphorylate Hippo components, LATS1 and LATS2 [30, 31]. In addition, it has been demonstrated that KIBRA can co-localize and interact with the PAR complex in mammalian epithelial cells [32, 33]. Based on these reports, we hypothesize that elevated expression of Par3 may inactivate the Hippo-YAP pathway via forming a complex with KIBRA to interfere its function on phosphorylation of the Hippo pathway.

**Fig. 5:**
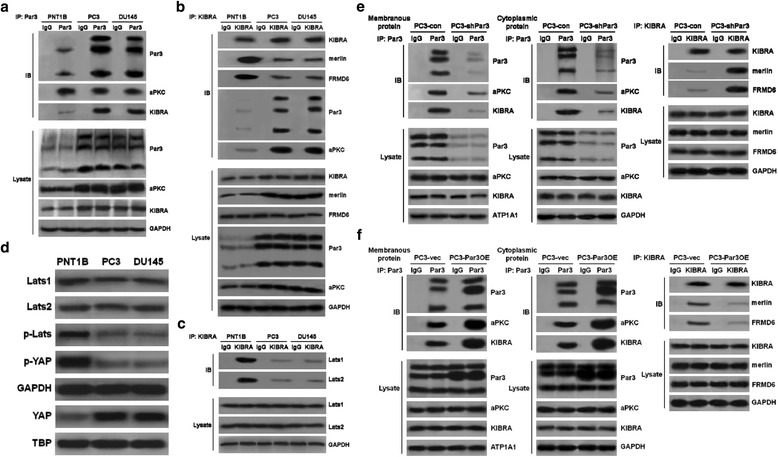
Elevated expression of Par3 dissociates KIBRA/Merlin/FRMD6 complex by increasing its interaction with KIBRA. **a** Endogenous interaction of Par3 with KIBRA is increased in both PC3 and DU145 cells compared to PNT1B cells. **b** Endogenous interaction of KIBRA with Merlin and FRMD6 is attenuated in both PC3 and DU145 cells compared to PNT1B cells. **c** Endogenous interaction of KIBRA with LATS1 and LATS2 is attenuated in both PC3 and DU145 cells compared to PNT1B cells. **d** Phosphorylation of LATS and YAP is decreased and the nuclear translocation of YAP is increased in both PC3 and DU145 cells compared to that in PNT1B cells. **e** Knockdown of Par3 decreases the interaction of both membranous and cytoplasmic Par3 with KIBRA and restores the interaction of KIBRA with Merlin and FRMD6. **f** Overexpression of Par3 further enhances the interaction of Par3 with KIBRA especially in cytoplasm and attenuates the interaction of KIBRA with Merlin and FRMD6

To test this possibility, a co-immunoprecipitation (co-IP) assay was carried out to determine potential interactions among KIBRA, the PAR complex and the Hippo components in non-tumorigenic PNT1B prostatic cells and PC3 and DU145 PCa cell lines respectively (Fig. 5a, b, c). Our results revealed that besides interaction with aPKC to form a canonical PAR complex in both normal prostatic and PCa cell lines, a non-canonical interaction of Par3 with KIBRA was significantly enhanced in PC3 and DU145 cells (Fig. 5a). Consistently, we observed an enhanced interaction of KIBRA with Par3 and aPKC and an attenuated interaction with its canonical partners Merlin and FRMD6 in both PC3 and DU145 cells but not PNT1B cells (Fig. 5b). Furthermore, although KIBRA was found to interact with LATS1/2 in all of the three cell lines, the interaction was significantly weakened in both PC3 and DU145 cells (Fig. 5c). As a result, the phosphorylation of both LATS and downstream YAP was repressed and the nuclear translocation of YAP was consequentially enhanced in both PC3 and DU145 cells (Fig. 5d). These results raised a possibility that elevated expression of Par3 may sequestrate KIBRA from its partners Merlin and FRMD6, and thus dissociate canonical KIBRA/Merlin/FRMD6 complex so that the Hippo pathway is inactivated due to impaired function of the KIBRA/Merlin/FRMD6 complex on the phosphorylation of LATS [30, 31]. In order to further investigate this possibility, we repeated the above co-IP assay in either Par3 knockdown (PC3-shPar3) or overexpression (PC3-Par3OE) PC3 cells to identify the sequestration of KIBRA by Par3 (Fig. 5e, f).

Considering that Par3 can be localized in both the cytoplasm and the membrane of cancer cells and that cytoplasmic Par3 may function differently from membranous Par3 [34], we carried out the following experiments. First, we determined expression levels of Par3 in both the cytoplasm and the membrane of PCa cell lines vs. control cells by IF staining. As shown in Figure S7a, an elevated expression of Par3 was observed in both the cytoplasm and the membrane in PC3 and DU145 cells compared to PNT1B cells (Additional file 2: Figure S7a). Similar results were obtained in 35 PCa clinical samples compared to normal samples from a tissue chip (HProA100PG01, Shanghai Outdo Biotech Co., data shown in Additional file 2: Figure S7b and Additional file 4: Table S1). Next, we separated membranous protein extraction from cytoplasmic protein extraction and checked the interaction of membranous or cytoplasmic Par3 with KIBRA respectively. As shown in Fig. 5e, knockdown of Par3 decreased the interaction of both membranous and cytoplasmic Par3 with KIBRA. As a response to the release of KIBRA from Par3, the formation of canonical KIBRA/Merlin/FRMD6 complex was restored (Fig. 5e). In contrast, overexpression of Par3 enhanced the sequestration of KIBRA by both membranous and cytoplasmic Par3 so that the interaction of KIBRA with Merlin and FRMD6 was further impaired (Fig. 5f). Therefore, the findings together indicate that both of membranous and cytoplasmic Par3 have the same function on the regulation of the Hippo pathway. They both can sequestrate KIBRA to impair the formation of the canonical KIBRA/Merlin/FRMD6 complex, which in turn causes a decrease of the phosphorylation of LATS and YAP to promote metastasis.

### Knockdown of Par3 decreases expression of pro-metastatic genes via impairing nuclear located YAP-mediated activation of TEAD transcription factors.

**Fig. 6:**
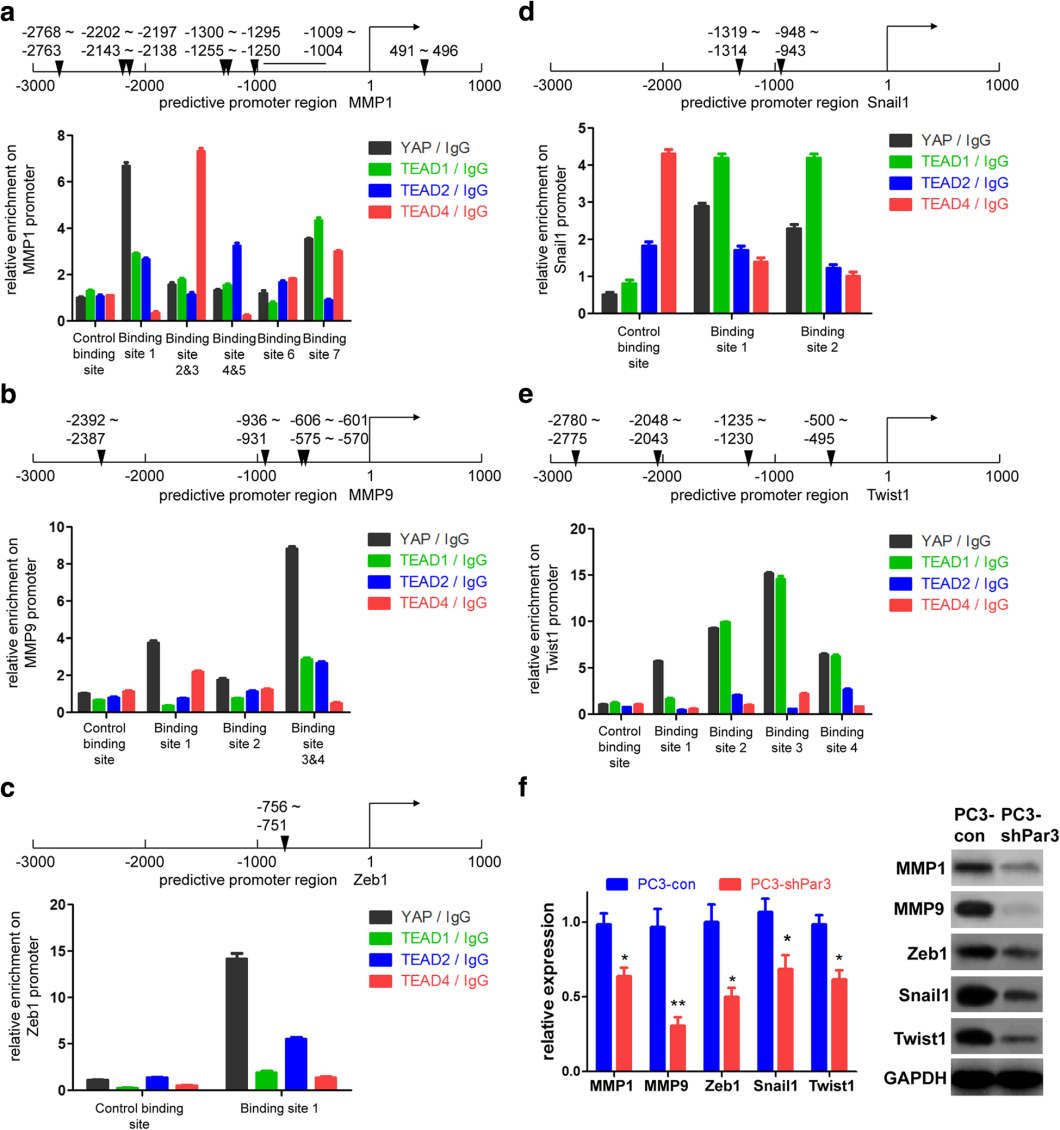
Knockdown of Par3 decreases the expression of pro-metastatic genes in PC3 cells. **a**-**e** Binding sites of TEAD transcription factors (mainly refer to TEAD1, 2 and 4) in the promoter region of MMP1 (**a**), MMP9 (**b**), Zeb1 (**c**), Snail1 (**d**) and Twist1 (**e**) respectively. The relative enrichment is significantly improved using either TEAD antibodies or a YAP antibody. A black triangle indicates binding site(s) for TEAD transcription factors. **f** Expression of MMP1/9, Zeb1, Snail1 and Twist1 is inhibited after Par3 knockdown at both mRNA and protein levels. All data are represented as mean ± SD from triplicate experiments. *: *p* < 0.05, **: *p* < 0.01

Given that Par3 promotes PCa metastasis via an inactivation of the Hippo pathway and a consequential enhancement of nuclear translocation of YAP by sequestration of KIBRA from its canonical KIBRA/Merlin/FRMD6 complex, we attempt to investigate potential downstream effectors of YAP, which are associated with metastasis. It has been known that YAP activates gene transcription by conjunction with the TEAD transcription factors family (mainly refer to TEAD1, 2 and 4, which share the same binding site) [14, 35–37]. Thus, we attempted to screen and identify potential TEAD binding sites by bioinformatics analysis using the PROMO online software [38] and chromatin immunoprecipitation (ChIP) assay in the promoter region of well-known pro-metastatic genes such as the matrix metalloproteinase (MMP) family, Zeb1, et al. We found that TEAD binding sites were harbored in the promoter region of MMP1, MMP9, Zeb1, Snail1 and Twist1 respectively (Fig. 6a-e), indicating that these pro-metastatic genes could be activated at the transcriptional level by YAP. Consistent with the results above, expression levels of these five genes was downregulted after Par3 knockdown, as a response to the decreased nuclear translocation of YAP (Fig. 6f, Additional file 2: Figure S8a). Conversely, opposite results were observed after Par3 overexpression in these cells (Additional file 2: Figure S8b). Collectively, the data demonstrate that elevated expression of Par3 increase expression of pro-metastatic genes via promotion of nuclear YAP-mediated activation of TEAD transcription factors.

## Discussion

Although loss or dislocalization of polarity proteins has been shown to be involved in tumorigenesis, whether and how it plays a role in tumor metastasis are unclear. The present study provides experimental evidence that not only a significant upregulation of Par3 is positively correlated with PCa, but also Par3 expression is further elevated in tissues from patients with metastasis compared to those without metastasis. In addition, our transwell assays in vitro as well as orthotopic implantation experiments in vivo verifies the functional effect of forced expression or knockdown of Par3 on the enhancement or reduction of PCa cell migration and tumor metastasis. Based on these findings, we propose that RNA or protein expression levels of Par3, measured by qRT-PCR, western blot or immunofluorescence, can be used to predict prostatic tumor metastasis, in combination with pathologic analyses.

While it is well-reported that destabilization of the polarity complex such as the PAR complex or loss of polarity proteins can induce EMT to promote tumor metastasis [5], the present experiments show that aberrantly elevated expression of Par3 also causes EMT. For example, previous studies indicate that loss of Par3 is reported to promote metastasis in breast cancer [5, 39] and esophageal squamous cell carcinoma [40]. Similarly, our previous work shows that knockdown of Shp2, a tyrosine phosphatase, can fasten the PAR complex without an influence on Par3 expression and weaken EMT to inhibit PCa metastasis [41]. The current work, on the other hand, shows an elevated expression of endogenous Par3 in PC3 and DU145 cells at both membranous and cytoplasmic locations as compared to non-tumorigenetic PNT1B prostatic epithelial cells. In addition, Par3 expression is more significantly upregulated in clinical metastatic PCa samples. Consistently, clinical studies by others in hepatocellular carcinomas [9], ovarian cancer [8] as well as clear cell renal cell carcinomas [34] demonstrated that Par3 acts as a positive regulator and is associated with a poor prognosis. More importantly, the present study showed that Par3 overexpression triggers expression of EMT-related genes such as Zeb1, Snail1, Twist1 and MMP1/9, by inactivation of metastasis-associated Hippo pathway [11]. These findings together suggest that maintaining an appropriate expression level of Par3 is important in prevention of tumor metastasis.

It is worth mentioning that previous studies only reported a positive or negative correlation between Par3 expression and metastasis in liver cancer [9] or in breast cancer [5], but fail to illustrate a clear mechanism underlying the metastasis induced by Par3 dysregulation. The present study reveals for the first time that elevated expression of Par3 promotes PCa metastasis via inactivation of the Hippo pathway mediated by forming a non-canonical Par3/aPKC/KIBRA complex as a definite mechanism. We demonstrate that besides formation of a canonical PAR complex, overexpression of Par3 can sequestrate KIBRA to form a non-canonical Par3/aPKC/KIBRA complex, resulting in a dissociation of the canonical KIBRA/Merlin/FRMD6 complex and a decrease of phosphorylation of LATS. Knockdown of Par3 restores phosphorylation of LATS to activate the Hippo pathway via dissociation of the Par3/aPKC/KIBRA complex to release KIBRA from this complex, resulting in phosphorylation of downstream YAP for a cytoplasmic arrest. Consequently, a decrease in nuclear translocation of YAP occurs, which triggers transcription of pro-metastatic genes. Our findings are consistent with a recent report showing that Par3 can promote interaction of PP1A (protein phosphatase 1 catalytic subunit alpha) with LATS1 to dephosphorylate LATS1 and YAP so that the nuclear translocation of YAP is increased to induce cell growth in HEK293T, A375 (a melanotic melanoma cell line) and T-47D (a breast cancer cell line) cells [42]. Support for our data also comes from another recent study that reveals a function of Par3 in the regulation of YAP phosphorylation [43]. Considered together, elevated expression of Par3, as a novel upstream regulator of the Hippo pathway, promotes PCa metastasis via inactivating the Hippo pathway.

Notably, based on ChIP assays, the current work has identified several putative downstream effectors of YAP for PCa metastasis. We demonstrate that decrease of nuclear translocation of YAP by knockdown of Par3 disrupts its conjunction with the TEAD transcription factors family, leading to the transcriptional suppression of multiple pro-metastatic genes including MMP1/9, Zeb1, Snail1 and Twist1, which play a role in EMT [44]. Collectively, these effectors identified in the present study expand the list of YAP target genes and also indicate that PCa metastasis induced by Par3 overexpression is associated with the upregulation of EMT-related genes. However, when and how Par3 expressional profiling and localization change during prostatic tumor metastasis remains to be determined.

## Conclusions

**Fig. 7:**
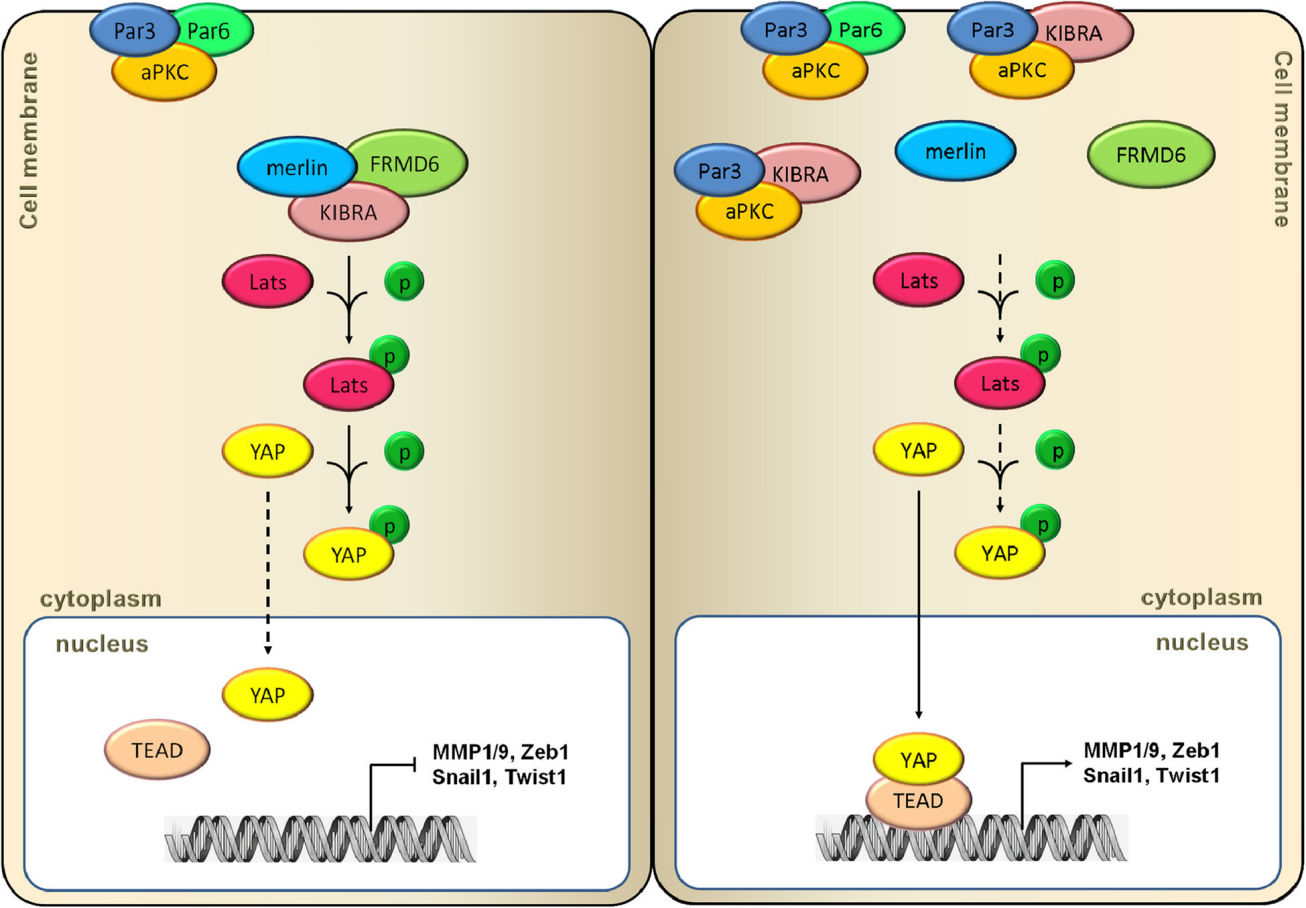
Elevated expression of Par3 promotes PCa metastasis via Hippo pathway inactivation. In PCa, Elevated expression of Par3 enhances its interaction with KIBRA to form a Par3/aPKC/KIBRA complex so to dissociate KIBRA/Merlin/FRMD6 complex. As a result, the interaction of KIBRA with LATS is attenuated and phosphorylation of LATS and downstream YAP is decreased to enhance the nuclear translocation of YAP, which promotes transcription of several pro-metastatic genes by conjunction of YAP with TEAD transcription factors. p: phosphorylation

Taken together, our study underscores the biological and clinical significance of Par3 in PCa. Elevated expression of Par3 promotes PCa metastasis via inactivation of the Hippo pathway in a KIBRA sequestration-dependent manner. Knockdown of Par3 dissociates the Par3/aPKC/KIBRA complex and activates the Hippo pathway, leading to a suppression of YAP-induced transcription of pro-metastatic factors (Fig. 7). Pharmaceutical intervention of Par3 or in combination with other classic therapeutic approaches might provide a more promising strategy to inhibit PCa metastasis.

## Additional files

Additional file 1: Table S1. qRT-PCR primers for this study. **Table S2.** Antibodies for this study. **Table S3.** qPCR primers of ChIP assay for this study. (PDF 56 kb)

Additional file 2: Figure S1. Par3 is upregulated in prostate cancer tissues. Data from TCGA PRAD database demonstrates a significant upregulation of Par3 expression in PCa tissues compared to normal controls. Data are represented as mean ± SD. *: *p* < 0.05 **Figure S2.** Par3 is upregulated in prostate cancer cell lines. (a) Endogenous expression of Par3 is tested by western blot. (b, c) stable knockdown of Par3 in PC3 (b) and DU145 (c) by Par3 specific shRNA respectively. (d) Expression level of Par3 is improved after infection of a lentiviral vector to overexpress a 150 kDa isoform of Par3. **Figure S3.** Knockdown of Par3 inhibits DU145 cell migration and invasion in vitro by transwell assays. Migratory cells are counted and averaged from five randomly selected fields. Scale Bar: 20 μm. All data are represented as mean ± SD from triplicate experiments. *: *p* < 0.05 **Figure S4.** Knockdown of Par3 inhibits prostate cancer invasion and metastasis in vivo. (a) Knockdown of Par3 inhibits invasion in peritoneal lymph nodes in PC3-shPar3 inoculated mice. (b) Representative PET-CT images for orthotopic implantation mouse models by inoculation of DU145-con cells (#1, #2, *n* = 2) or DU145-shPar3 cells (#3, #4, *n* = 2). (c) Orthotopic grafts and lymph nodes invasion are dramatically regressed in DU145-shPar3 inoculated mouse (#3, #4) than control (#1, #2). **Figure S5.** Knockdown of Par3 decreases while overexpression of Par3 increases the nuclear translocation of oncogene YAP. (a) Overespression of Par3 in PC3 cells suppresses the phosphorylation of LATS and YAP so to enhance the nuclear translocation of YAP. (b) Knockdown of Par3 in DU145 cells enhances the phosphorylation of LATS and YAP and decreases nuclear translocation of YAP. (c) IF staining of nuclear translocation of YAP in DU145-con and DU145-shPar3 cells respectively in vitro. Scale Bar: 20 μm. **Figure S6.** Inhibition of PCa metastasis by Par3 knockdown is reversed after overexpression of YAP(S127A). (a) Overexpression of a non-phosphorylated YAP mutant YAP(S127A) enhances nuclear translocation of YAP. (b) Representative PET-CT images from orthotopic implantation mouse models by inoculation of PC3-shPar3-vec or PC3-shPar3-YAP(S127A) cells. (c) Orthotopic grafts and liver metastasis are restored in PC3-shPar3-YAP(S127A) inoculated mouse but not in control. (d) H&E staining of liver tissues from orthotopic PC3-shPar3-vec or PC3-shPar3-YAP(S127A) cells inoculated mice. Field in frame: metastatic nodes. Scale Bar: 100 μm for upper panels; 50 μm for lower panels. (e) IF staining of liver tissues for YAP expression from orthotopic PC3-shPar3-vec or PC3-shPar3-YAP(S127A) cells inoculated mice. Field in white broken line: metastatic nodes in liver. H-nuclei: human nuclei. Scale Bar: 20 μm. **Figure S7.** Elevated expression of Par3 is detected at both membrane and cytoplasm. (a) Overxpression of Par3 is identified with both membranous and cytoplasmic location in PC3 and DU145 cells. Scale Bar: 20 μm (b) Representative images of Par3 expression in normal control and prostate cancer tissues from Gleason Score (GS) 7 to 9 patients. Scale Bar: 100 μm for panels in the first column from left; 20 μm for panels in other three columns. **Figure S8.** Knockdown of Par3 decreases while overexpression of Par3 increases expression of pro-metastatic genes. (a) Expression of MMP1/9, Zeb1, Snail1 and Twist1 is suppressed by Par3 knockdown in DU145 cells at both mRNA and protein levels. (b) Expression of MMP1/9, Zeb1, Snail1 and Twist1 is improved by Par3 overexpression in PC3 cells at both mRNA and protein levels. All data are represented as mean ± SD from triplicate experiments. *: p < 0.05, **: *p* < 0.01 (PDF 2909 kb)

Additional file 3: **Table S1.** Clinical data from 14 patients and 7 normal persons for qRT-PCR and western blot assay in the study. **Table S2.** Clinical data from 2 patients and 1 normal person for IHC staining in the study. (PDF 48 kb)

Additional file 4: Table S1. Clinical data of 35 PCa patient samples from a tissue chip. (PDF 52 kb)

## Abbreviations

aPKC: Atypical protein kinase C
ChIP: Chromatin immunoprecipitation
co-IP: Co-immunoprecipitation
CTGF: Connective tissue growth factor
GPCR: G protein-coupled receptor
IF: Immunofluorescence
IHC: Immunohistochemical
LATS1/2: Large tumor suppressor kinase 1/2
MMP: Matrix metalloproteinase
PAR complex: Partitioning defective complex
PCa: Prostate cancer
TEAD: TEA domain
YAP: Yes-associated protein
