# Supplementary material for: Elevated expression of Par3 promotes prostate cancer metastasis by forming a Par3/aPKC/KIBRA complex and inactivating the hippo pathway
Source: J Exp Clin Cancer Res. 2017 Oct 10;36:139. doi: 10.1186/s13046-017-0609-y (PMC5633884; doi:10.1186/s13046-017-0609-y)
Supplement: Supplementary file 1 — qRT-PCR primers for this study. Table S2. Antibodies for this study. Table S3. qPCR primers of ChIP assay for this study. (PDF 56 kb) [file 13046_2017_609_MOESM1_ESM.pdf]

Additional file 1:
Table S1: qRT-PCR primers for this study.

Name	Sequence (5' - 3')
Par3 (F)	TATAATGCGAGGGTCTGGACGG
Par3 (R)	CGTCTAAGCAGCTGCAAGGAG
MMP1 (F)	GGTTGCGCTTTCCTTTCTGT
MMP1 (R)	CTTTCTCCGCAGTTTCCTCA
MMP9 (F)	GGGCAATCAATGGATGAAAG
MMP9 (R)	AGTAATTCTTCACCCTGCAG
Snail1 (F)	TATAATGCGAGGGTCTGGACGG
Snail1 (R)	CGTCTAAGCAGCTGCAAGGAG
Twist1 (F)	TAAACTGGGGTCGCATTGTG
Twist1 (R)	GGTGGTCATTCAGGTAAGTG
Zeb1 (F)	GGTTGCGCTTTCCTTTCTGT
Zeb1 (R)	CTTTCTCCGCAGTTTCCTCA

F: forward primer, R: reverse primer

Table S2: Antibodies for this study.

Name	Company	Lot No.
Par3	Millipore	07-330
aPKC	Santa cruz	sc-216
KIBRA	Cell Signaling Technology	#8774
Merlin	Cell Signaling Technology	12888
FRMD6	Santa cruz	sc-138008
Lats1	Cell Signaling Technology	#3477
Lats2	Cell Signaling Technology	#5888
p-Lats	Cell Signaling Technology	#8654
YAP	Cell Signaling Technology	#4912
p-YAP	Cell Signaling Technology	#4911
MMP1	Santa cruz	sc-21731
MMP9	Santa cruz	sc-6840
Zeb1	Santa cruz	sc-10572
Snail1	Cell Signaling Technology	#3879
Twist1	Santa cruz	sc-15393
ATP1A1[a]	Santa cruz	sc-16041
TBP[b]	Proteintech	66166-1g
GAPDH	Cell Signaling Technology	#8884

a: ATP1A1: ATPase Na^+/K^+ transporting subunit alpha 1, an internal control for membranous protein

b: TBP, TATA-box binding protein, an internal control for nuclear protein

Table S3: qPCR primers of ChIP assay for this study.

Name		Sequence (5' - 3')
MMP1	Site 1	GCTTTGAGGCCTTTCACAGA (F)<br>CCTTTACCCCTTATGACAAG (R)
	Site 2	TGAGCATTTAGCATGAGAGC (F)<br>GAAATAGAGCCTTGGAGTCC (R)
	Site 3	ACAGGCCAGTGTTTCTCCA (F)<br>GGTGTCACCAGTGCTATCT (R)
	Site 4	TAGCACCAAGGAGCGAAGAT (F)<br>CCTTCCCTGAAACTTGTCTG (R)
	Site 5	AGTTGGAGGTGAGAGTGAAC (F)<br>TCGCAGTTGCTTTTATCCTT (R)
	Contrl site	AACAAAGGCAGAAGGGAACC (F)<br>GGAATTACTAACACTGCGCAC (R)
MMP9	Site 1	ACCCAGATGAAGATCCTCCA (F)<br>GCATTTAGGGAATCAGAGGC (R)
	Site 2	GCCTCCCAAAGTGCTAAGAT (F)<br>CTGTAGGTTGTAAGTCCGCA (R)
	Site 3	TTCTAGAGGCTGCTACTGTC (F)<br>GCCTTCTTTGACTCAGCTTC (R)
	Contrl site	CCTTTCTCATGCTGGTGCTG (F)<br>CTGACTGCAGCTGCTGTTGT (R)
Zeb1	Site 1	GTGGGTATTACTCATTCCGC (F)<br>GGGATGGGAAGTGACTTCAA (R)
	Contrl site	AAGGGAGGAAGAACTCACAC (F)<br>CAGTCCTCGCTTTCCTTGAT (R)
Snail1	Site 1	GGCACCAAGTGACTAAACAG (F)<br>TCACAGGTCTCACCGTTCTT (R)
	Site 2	TGAGGCAAAGTCCAAACTCC (F)<br>GGCAACTGCTTCAGAGTAAC (R)
	Contrl site	TACAGGATTGCACCAGGGTT (F)<br>AACTGTTGTCCAGCCCAAAC (R)
Twist1	Site 1	GAGCCATCCTTGCAAATGTG (F)<br>ACTCCCTGCTTTGCAAACTG (R)
	Site 2	TGGCCACAAAGGAAAGAGTC (F)<br>GCTGCAGAAATCTCGAAACC (R)
	Site 3	GGATGGACCTGAAACGGTTA (F)<br>AGCAGCAAAGTTGGTGAACC (R)
	Site 4	GTTCGTCTACCTGACCATTG (F)<br>CCGATTTGGGATTGCTGG (R)
	Contrl site	GGACAAGCTGAGCAAGATTC (F)<br>ACATAGCTGCAGCTTGCCAT (R)

F: forward primer, R: reverse primer
