# Supplementary material for: Elevated expression of Par3 promotes prostate cancer metastasis by forming a Par3/aPKC/KIBRA complex and inactivating the hippo pathway
Source: J Exp Clin Cancer Res. 2017 Oct 10;36:139. doi: 10.1186/s13046-017-0609-y (PMC5633884; doi:10.1186/s13046-017-0609-y)
Supplement: Supplementary file 2 — Par3 is upregulated in prostate cancer tissues. Data from TCGA PRAD database demonstrates a significant upregulation of Par3 expression in PCa tissues compared to normal controls. Data are represented as mean ± SD. *: p < 0.05 Figure S2. Par3 is upregulated in prostate cancer cell lines. (a) Endogenous expression of Par3 is tested by western blot. (b, c) stable knockdown of Par3 in PC3 (b) and DU145 (c) by Par3 specific shRNA respectively. (d) Expression level of Par3 is improved after infection of a lentiviral vector to overexpress a 150 kDa isoform of Par3. Figure S3. Knockdown of Par3 inhibits DU145 cell migration and invasion in vitro by transwell assays. Migratory cells are counted and averaged from five randomly selected fields. Scale Bar: 20 μm. All data are represented as mean ± SD from triplicate experiments. *: p < 0.05 Figure S4. Knockdown of Par3 inhibits prostate cancer invasion and metastasis in vivo. (a) Knockdown of Par3 inhibits invasion in peritoneal lymph nodes in PC3-shPar3 inoculated mice. (b) Representative PET-CT images for orthotopic implantation mouse models by inoculation of DU145-con cells (#1, #2, n = 2) or DU145-shPar3 cells (#3, #4, n = 2). (c) Orthotopic grafts and lymph nodes invasion are dramatically regressed in DU145-shPar3 inoculated mouse (#3, #4) than control (#1, #2). Figure S5. Knockdown of Par3 decreases while overexpression of Par3 increases the nuclear translocation of oncogene YAP. (a) Overespression of Par3 in PC3 cells suppresses the phosphorylation of LATS and YAP so to enhance the nuclear translocation of YAP. (b) Knockdown of Par3 in DU145 cells enhances the phosphorylation of LATS and YAP and decreases nuclear translocation of YAP. (c) IF staining of nuclear translocation of YAP in DU145-con and DU145-shPar3 cells respectively in vitro. Scale Bar: 20 μm. Figure S6. Inhibition of PCa metastasis by Par3 knockdown is reversed after overexpression of YAP(S127A). (a) Overexpression of a non-phosphorylated YAP mutant YAP(S [file 13046_2017_609_MOESM2_ESM.pdf]

Additional file 2:

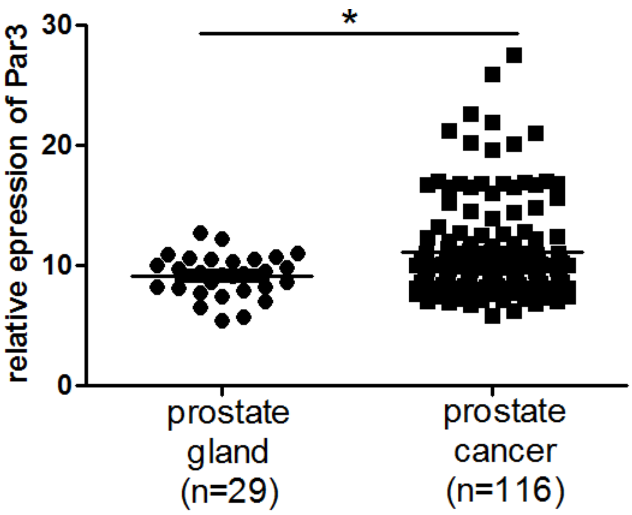

Figure S1. Par3 is upregulated in prostate cancer tissues. Data from TCGA PRAD database demonstrates a significant upregulation of Par3 expression in PCa tissues compared to normal controls. Data are represented as mean ± SD. ＊: $p<0.05$

Relevant data can be obtained by the following link:

https://portal.gdc.cancer.gov/exploration?filters=~%28op~%27and~content~%28~%28op~%27in~content~%28field~%27cases.primary_site~value~%28~%27Prostate%29%29%29%29%29%29

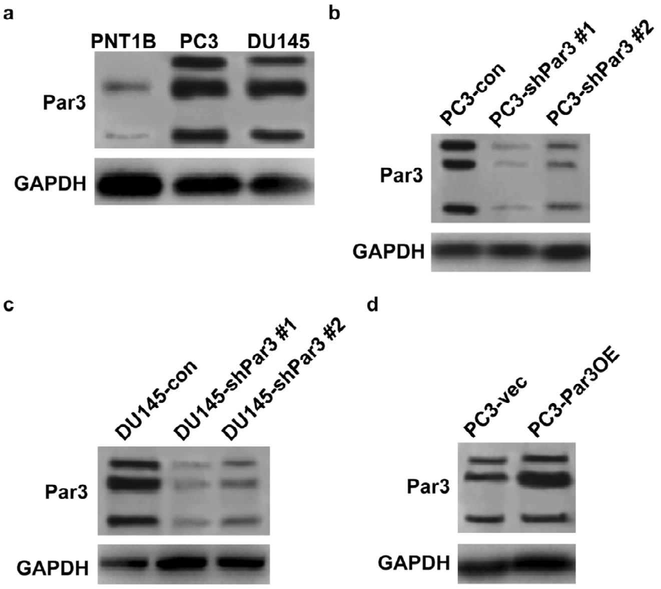

Figure S2. Par3 is upregulated in prostate cancer cell lines. (a) Endogenous expression of Par3 is significantly enhanced in prostate cancer cell lines PC3 and DU145 than that in normal prostatic epithelial cell line PNT1B by western blot. (b, c) Par3 expression can be suppressed by two independent vectors which contain different Par3 shRNA (#1 or #2) respectively. Because stronger effect on Par3 suppression can be obtained at protein level when using shPar3#1, the vector containing shPar3#1 is selected to establish a subclone for stable Par3 knockdown in PC3 (b) and DU145 (c) respectively. (d) Expression level of Par3 is improved after infection of a lentiviral vector to overexpress a 150kDa isoform of Par3.

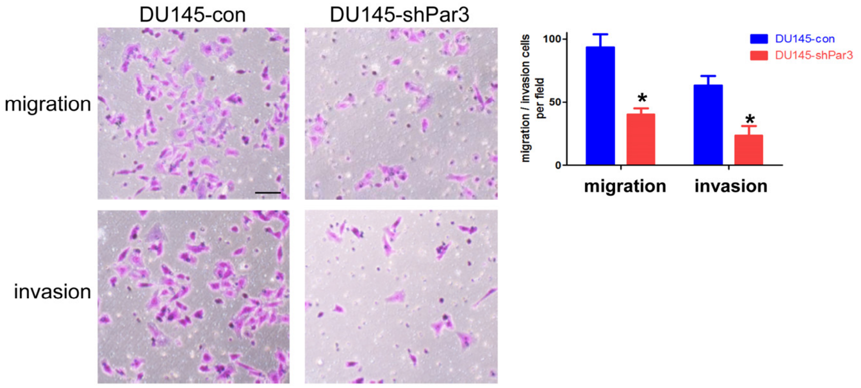

Figure S3. Knockdown of Par3 inhibits DU145 cell migration and invasion *in vitro* by transwell assays. Migratory cells are counted and averaged from five randomly selected fields. Scale Bar: 20μm. All data are represented as mean ± SD from triplicate experiments. ⋆: $p<0.05$

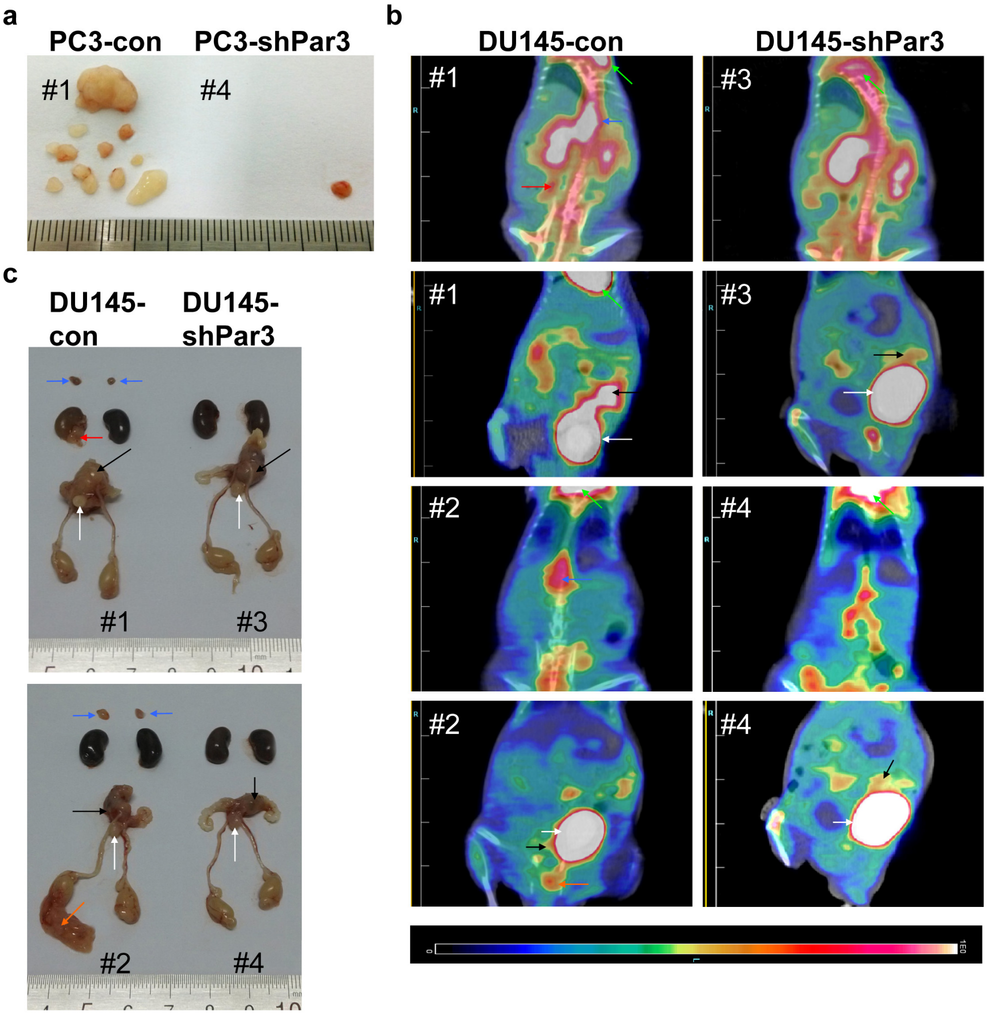

Figure S4. Knockdown of Par3 inhibits prostate cancer invasion and metastasis *in vivo*. (a) Knockdown of Par3 inhibits invasion in peritoneal lymph nodes in PC3-shPar3 inoculated mice. (b) Representative PET-CT images for orthotopic implantation mouse models by inoculation of DU145-con cells (#1, #2, n=2) or DU145-shPar3 cells (#3, #4, n=2). White arrow: bladder, black arrow: orthotopic tumor, blue arrow: invasion in paravertebral lymph node, red arrow: metastasis in renal pelvis, orange arrow: fatty cyst in testis, bright green: heart. (c) Orthotopic grafts and lymph nodes invasion are dramatically regressed in DU145-shPar3 inoculated mouse (#3, #4) than control (#1, #2). White arrow: bladder, black arrow: orthotopic tumor, blue arrow: invasion in paravertebral lymph node, red arrow: metastasis in renal pelvis, orange arrow: fatty cyst in testis.

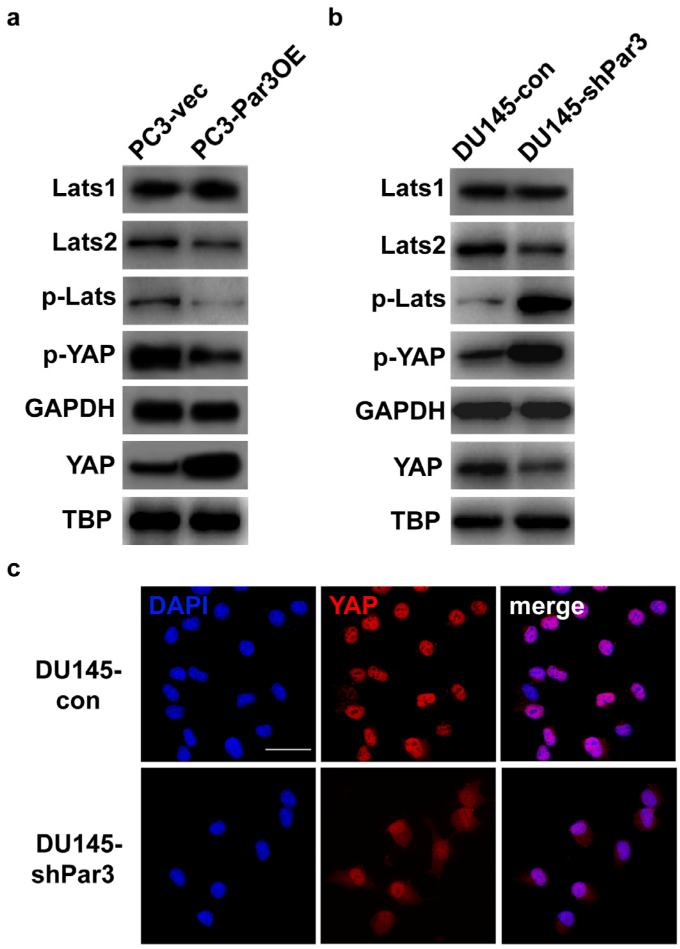

Figure S5. Knockdown of Par3 decreases while overexpression of Par3 increases the nuclear translocation of oncogene YAP. (a) Overespression of Par3 in PC3 cells suppresses the phosphorylation of LATS and YAP so to enhance the nuclear translocation of YAP. GAPDH: internal control for cytoplasmic proteins; TBP: internal control for nuclear proteins. (b) Knockdown of Par3 in DU145 cells enhances the phosphorylation of LATS and YAP and decreases nuclear translocation of YAP. GAPDH: internal control for cytoplasmic protein; TBP: internal control for nuclear protein. (c) IF staining of nuclear translocation of YAP in DU145-con and DU145-shPar3 cells respectively *in vitro*. Scale Bar: 20μm.

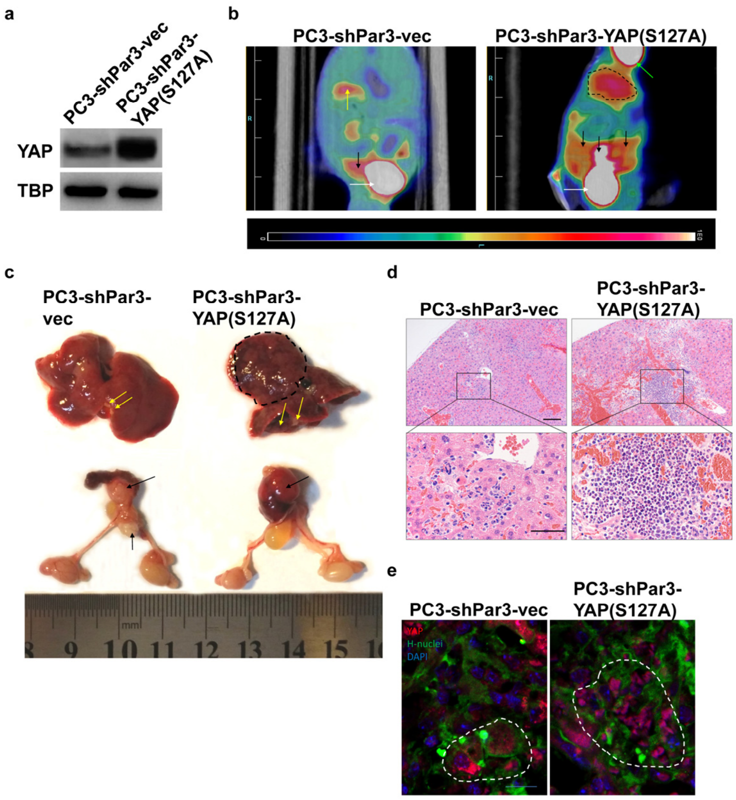

Figure S6. Inhibition of PCa metastasis by Par3 knockdown is reversed after overexpression of YAP(S127A). (a) Overexpression of a non-phosphorylated YAP mutant YAP(S127A) enhances nuclear translocation of YAP. GAPDH: internal control for cytoplasmic protein; TBP: internal control for nuclear protein. (b) Representative PET-CT images from orthotopic implantation mouse models by inoculation of PC3-shPar3-vec or PC3-shPar3-YAP(S127A) cells. White arrow: bladder, black arrow: orthotopic tumor, yellow arrow and field in black broken line: metastatic nodes in liver, bright green: heart. (c) Orthotopic grafts and liver metastasis are restored in PC3-shPar3-YAP(S127A) inoculated mouse but not in control. Black arrow: orthotopic tumor, yellow arrow and field in black broken line: metastatic nodes in liver. (d) H&E staining of liver tissues from orthotopic PC3-shPar3-vec or PC3-shPar3-YAP(S127A) cells inoculated mice. Field in frame: metastatic nodes. Scale Bar: 100μm for upper panels; 50μm for lower panels. (e) IF staining of liver tissues for YAP expression from orthotopic PC3-shPar3-vec or PC3-shPar3-YAP(S127A) cells inoculated mice. Field in white broken line: metastatic nodes in liver. H-nuclei: human nuclei. Scale Bar: 20μm.

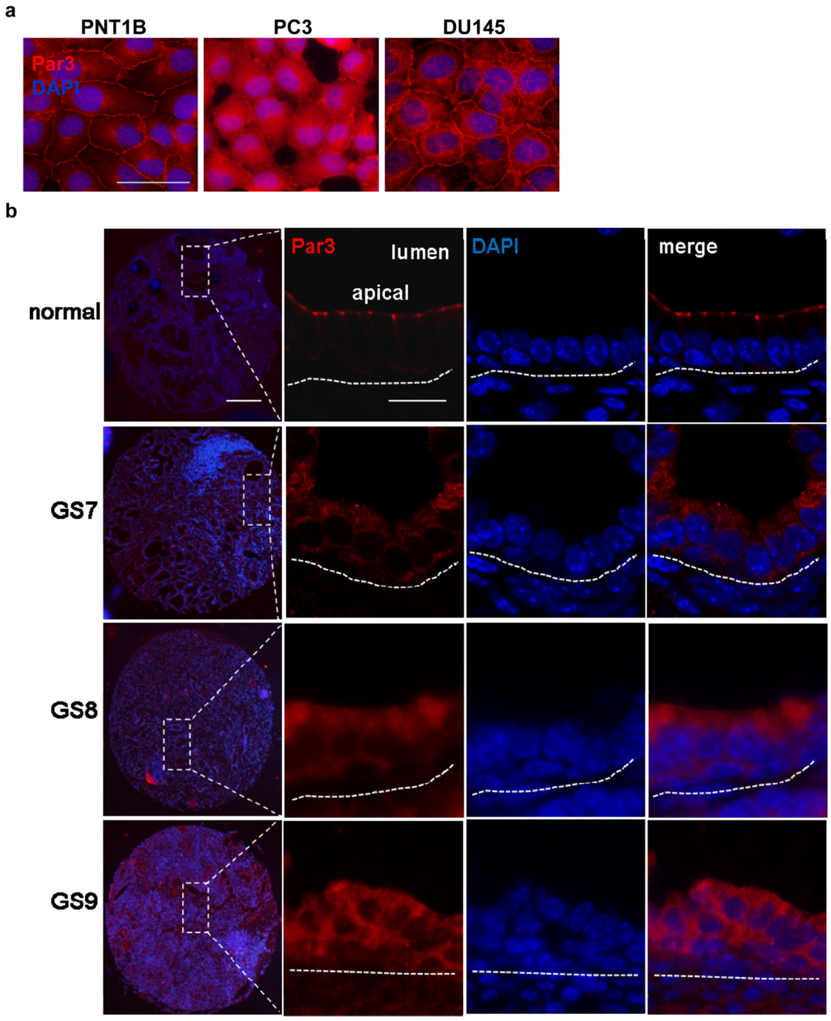

Figure S7. Elevated expression of Par3 is detected at both membrane and cytoplasm. (a) Overexpression of Par3 is identified with both membrane and cytoplasm location in PC3 and DU145 cells. Scale Bar: 20μm (b) Representative images of Par3 expression in normal control and prostate cancer tissues from Gleason Score (GS) 7 to 9 patients. Scale Bar: 100μm for panels in the first column from left; 20μm for panels in other three columns. Dotted line is used for separation of two adjacent lumens.

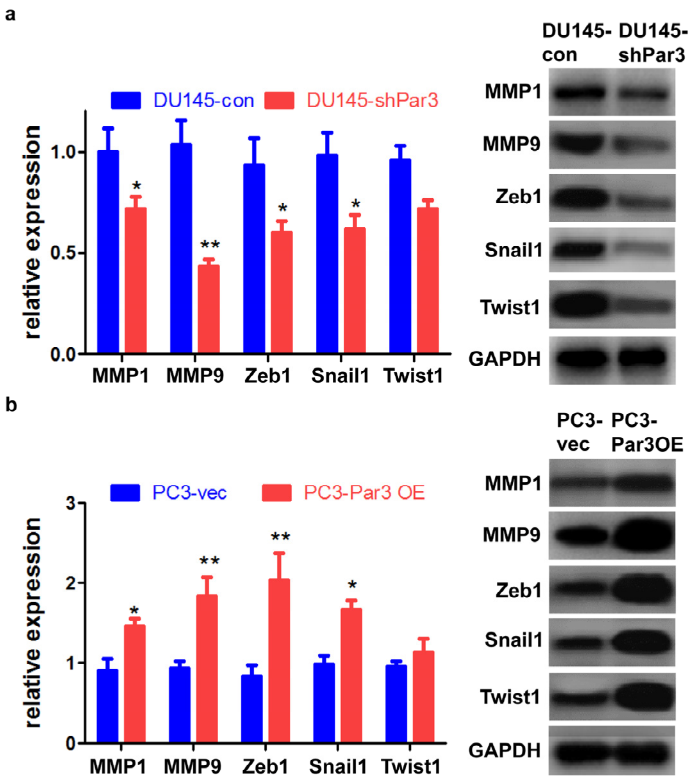

Figure S8. Knockdown of Par3 decreases while overexpression of Par3 increases expression of pro-metastatic genes. (a) Expression of MMP1/9, Zeb1, Snail1 and Twist1 is suppressed by Par3 knockdown in DU145 cells at both mRNA and protein levels. (b) Expression of MMP1/9, Zeb1, Snail1 and Twist1 is improved by Par3 overexpression in PC3 cells at both mRNA and protein levels. All data are represented as mean ± SD from triplicate experiments. ⋆: $p<0.05$, ⋆⋆: $p<0.01$
