# Supplementary material for: Elevated expression of Par3 promotes prostate cancer metastasis by forming a Par3/aPKC/KIBRA complex and inactivating the hippo pathway
Source: J Exp Clin Cancer Res. 2017 Oct 10;36:139. doi: 10.1186/s13046-017-0609-y (PMC5633884; doi:10.1186/s13046-017-0609-y)
Supplement: Supplementary file 3 — Table S1. Clinical data from 14 patients and 7 normal persons for qRT-PCR and western blot assay in the study. Table S2. Clinical data from 2 patients and 1 normal person for IHC staining in the study. (PDF 48 kb) [file 13046_2017_609_MOESM3_ESM.pdf]

Additional file 3:

**Table S1:** Clinical data from 14 patients and 7 normal persons for qRT-PCR and western blot assay in the study.

| | Age | Gleason Scores | TNM stage | Metastasis |
|---|---|---|---|---|
| N1 | 72 | — | — | — |
| N2 | 59 | — | — | — |
| N3 | 62 | — | — | — |
| N4 | 61 | — | — | — |
| N5 | 68 | — | — | — |
| N6 | 70 | — | — | — |
| N7 | 60 | — | — | — |
| P1 | 74 | 6 | T2bN0M0 | No |
| P2 | 57 | 7 | T2bN0M0 | No |
| P3 | 66 | 7 | T2cN0M0 | No |
| P4 | 72 | 7 | T2cN0M0 | No |
| P5 | 60 | 7 | T2aN0M0 | No |
| P6 | 73 | 7 | T2aN0M0 | No |
| P7 | 59 | 7 | T3bN0M1 | Yes |
| P8 | 62 | 7 | T3bN0M1 | Yes |
| P9 | 59 | 8 | T2cN0M1 | Yes |
| P10 | 77 | 8 | T4N2M1b | Yes |
| P11 | 72 | 8 | T3bN1M1 | Yes |
| P12 | 75 | 8 | T4N1M1 | Yes |
| P13 | 80 | 9 | T4N1M1 | Yes |
| P14 | 79 | 10 | T4N1M1 | Yes |

N: normal control; P: patient; —: Data not detected.

**Table S2:** Clinical data from 2 patients and 1 normal person for IHC staining in the study.

| | Age | Gleason Scores | TNM stage | Metastasis |
|---|---|---|---|---|
| Normal control | 71 | — | — | — |
| Prostate cancer without metastasis | 73 | 8 | T2cN1M0 | No |
| Prostate cancer with metastasis | 69 | 9 | T4N1M1 | Yes |

—: Data not detected.
