# Supplementary material for: Elevated expression of Par3 promotes prostate cancer metastasis by forming a Par3/aPKC/KIBRA complex and inactivating the hippo pathway
Source: J Exp Clin Cancer Res. 2017 Oct 10;36:139. doi: 10.1186/s13046-017-0609-y (PMC5633884; doi:10.1186/s13046-017-0609-y)
Supplement: Supplementary file 4 — Clinical data of 35 PCa patient samples from a tissue chip. (PDF 52 kb) [file 13046_2017_609_MOESM4_ESM.pdf]

Additional file 4:

Table S1: Clinical data of 35 PCa patient samples from a tissue chip.

	Tissue ID	Age	Gleason Score
P1	I05A1029	73	6
P2	I05A0919	74	6
P3	I05A1035	77	6
P4	I05A0925	75	6
P5	I05A1041	63	6
P6	I05A0928	72	6
P7	I05A0977	59	6
P8	I05A0990	67	6
P9	I05A1005	59	6
P10	I05A1017	64	6
P11	I05A1019	74	6
P12	I05A1023	67	6
P13#	I05A0917	77	7
P14	I05A1033	67	7
P15	I05A0961	73	7
P16	I05A0962	53	7
P17	I05A0964	66	7
P18	I05A0973	57	7
P19	I05A0980	70	7
P20	I05A0984	74	7
P21	I05A0987	73	7
P22	I05A1001	62	7
P23	I05A0981	52	7
P24	I05A1007	71	7
P25	I05A1008	58	7
P26	I05A1012	66	7
P27	I05A1014	69	7
P28	I05A1016	62	7
P29	I05A0965	65	8
P30#	I05A0978	70	8
P31	I05A0979	65	8
P32	I05A0993	74	8
P33	I05A1027	84	8
P34#	I05A1044	67	9
P35	I05A0976	69	9

The chip was purchased from Shanghai Outdo Biotech Company (http://www.outdobiotech.com) with chip ID HProA100PG01.

#A representative image shown in Figure S6b was observed by IF staining using tissues from patients labeled in #.
